# Pressure-driven formation and stabilization of superconductive chromium hydrides

**DOI:** 10.1038/srep17764

**Published:** 2015-12-02

**Authors:** Shuyin Yu, Xiaojing Jia, Gilles Frapper, Duan Li, Artem R. Oganov, Qingfeng Zeng, Litong Zhang

**Affiliations:** 1 Science and Technology on Thermostructural Composite Materials Laboratory, School of Materials Science and Engineering, Northwestern Polytechnical University, Xi’an, Shaanxi 710072, PR China; 2 International Center for Materials Discovery, School of Materials Science and Engineering, Northwestern Polytechnical University, Xi’an, Shaanxi 710072, PR China; 3 IC2MP UMR 7285, Université de Poitiers-CNRS, 4, rue Michel Brunet TSA 51106-86073 Poitiers Cedex 9, France; 4 Skolkovo Institute of Science and Technology, 5 Nobel Street, Skolkovo 143025, Russia; 5 Department of Geosciences, Center for Materials by Design, and Institute for Advanced Computational Science, State University of New York, Stony Brook, NY 11794-2100, USA; 6 Moscow Institute of Physics and Technology, Dolgoprudny, Moscow Region 141700, Russia

## Abstract

Chromium hydride is a prototype stoichiometric transition metal hydride. The phase diagram of Cr-H system at high pressures remains largely unexplored due to the challenges in dealing with the high activation barriers and complications in handing hydrogen under pressure. We have performed an extensive structural study on Cr-H system at pressure range 0 ∼ 300 GPa using an unbiased structure prediction method based on evolutionary algorithm. Upon compression, a number of hydrides are predicted to become stable in the excess hydrogen environment and these have compositions of Cr_2_H_*n*_ (n = 2–4, 6, 8, 16). Cr_2_H_3_, CrH_2_ and Cr_2_H_5_ structures are versions of the perfect anti-NiAs-type CrH with ordered tetrahedral interstitial sites filled by H atoms. CrH_3_ and CrH_4_ exhibit host-guest structural characteristics. In CrH_8_, H_2_ units are also identified. Our study unravels that CrH is a superconductor at atmospheric pressure with an estimated transition temperature (*T _c_*) of 10.6 K, and superconductivity in CrH_3_ is enhanced by the metallic hydrogen sublattice with *T _c_* of 37.1 K at 81 GPa, very similar to the extensively studied MgB_2_.

Since molecular organometallic complexes HCo(CO)_4_ and H_2_Fe(CO)_4_ were first synthesized in 1930’s^1^, almost all other transition metal hydrides have been successfully synthesized. The hydrogen ligand presents a large variety of coordination modes in cluster chemistry: terminal, bridging, capping or even interstitial positions. However, hydrogen atoms dissolved within bulk transition metals are relatively inert at ambient pressure. Only a few 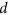-block transition metals exhibit stoichiometric or near-stoichiometric compositions, while most transition metals form non-stoichiometric binary hydrides in which hydrogen atoms are incorporated into the metal host lattice sites^2^ or interstitial sites^3^. In the transition metal family, chromium has been known to human beings for over 2000 years, dating back to the Chinese Qin dynasty when it was used by Chinese blacksmiths to coat metal weapons to prevent corrosion; such items have been discovered with the majestic Terracotta Army^4^.

During recent decades, discrete molecules made of chromium and hydrogen have been widely studied. In fact, these molecules have attracted a great deal of attention from both the experimental and theoretical communities due to their unusual reactivity and potential as homogeneous catalysts for hydrogenation and other reactions of organic substrates. Molecular CrH_2_ was first identified by Weltner *et al.* via electron spin-resonance spectroscopy (ESR) and IR spectroscopy in 1979^5^. Later, Margrave *et al.* reported the IR spectra of CrH_2_ in krypton and argon matrices^6^. Molecular CrH_3_ has also been identified in inert gas matrices using the photochemical reaction of chromium with hydrogen^7^. In 2003, Andrews *et al.* observed CrH, CrH_2_, (H_2_)CrH, (H_2_)CrH_2_ and (H_2_)_2_CrH_2_ complexes in solid argon, neon, hydrogen and deuterium matrices^8^. Gagliardi *et al.* predicted that chromium can constantly absorb hydrogen to form CrH_12_ molecules^9^. However, all of these substances are in the form of molecular species.

In the solid state, only a small number of studies have investigated chromium hydrides. As a typical first-row transition metal, chromium exothermically absorbs hydrogen into the interstitial sites to form the stoichiometric anti-NiAs-type CrH at ambient conditions; its corresponding isoelectronic analogues MoH^10^ and WH^11^ also crystallize in this structure at low pressures. In the past decades, many works focused on magnetic properties of the sub-stoichiometric CrH_0.97_. An intense debate on the magnetic state of CrH_0.97_ was finally resolved^12^. The material was found to be non-magnetic, and previous reports of its magnetism were related to magnetic impurities appearing during synthesis. The crystal structure of CrH_2_ has been reported to have cubic fluorite-type structure. However, there is no definitive crystallographic characterization of chromium dihydride or trihydride^13^,^14^,^15^,^16^.

At atmospheric pressure, the H
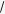
Cr ratio is small, i.e., lower or equal to 3, but one can expect a higher concentration of hydrogen in chromium when pressure increases. Tungsten hydrides with stoichiometries up to 1:8 have been recently reported^11^. The successful syntheses of NiH^17^, WH_*x*_^11^ and IrH_*x*_^3^ etc. via the diamond-anvil cell technique^18^ give to materials research community the opportunity to search for novel bulk Cr_*x*_H_*y*_ alloys at very high pressures (0 ∼ 300 GPa). Here, we apply recently evolutionary algorithm USPEX to extensively explore the crystal structures of Cr-H compounds, and then analyzed their electronic properties, chemical bonding and potential superconductivity.

## Results and Discussion

### Phase stability and structural characteristics of the Cr_*x*_H_*y*_ compounds

**Figure 1:**
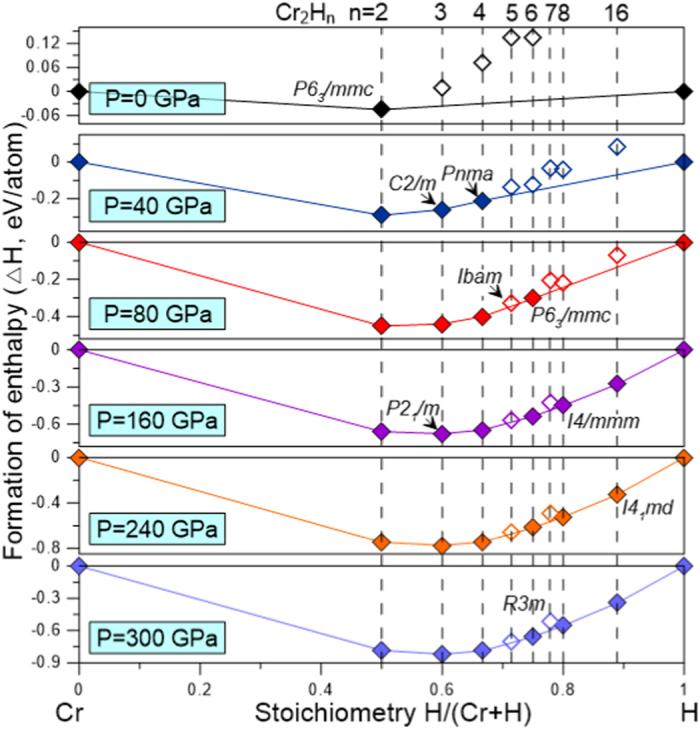
The calculated enthalpies of formation (ΔH_*f*_, per atom) including zero-point energy correction for Cr-H compounds at selected pressures.

In order to determine which chromium hydrides are thermodynamically stable at a given pressure, ground-state enthalpies of formation of the Cr_*x*_H_*y*_ phases have been calculated with respect to elemental chromium and hydrogen in their most stable forms, i.e., 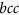 (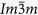) structure for Cr^15^, in the whole pressure range, and depending on the applied pressure, 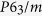, 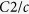 and 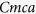-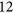 structures for hydrogen^19^. Fig. 1 summarizes our findings while more complete diagrams including metastable phases are given in SM Fig. S1, taking in account the vibrational contributions (zero-point energy) to the enthalpies for each Cr_*x*_H_*y*_, pure Cr and H_2_. As illustrated in Fig. 1, pressure stabilizes chromium hydrides. At 0 GPa, the anti-NiAs type CrH with the space group 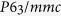 is the only thermodynamically stable structure, which is consistent with the experimental results. With increasing pressure, progressively hydrogen-richer compounds become stable, and their compositions can be represented as series Cr_2_H_*n*_ (n = 2–8, 16). As solid-state chromium is experimentally known to be magnetic at ambient pressure, we have looked at the effect of magnetism on the relative phase stability of chromium hydrides. Our main results show that all chromium hydrides are non-magnetic at ambient and high pressures, and that the magnetism correction did not affect the identification of the phases or their relative stability (see SM Tab. S1).

Based on an electron counting rule within ionic model (Cr^6+^, 6H^−^), we would like to recall that Cr has 6 valence electrons (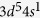), a number sufficient to reduce 3H_2_ to 6H^−^. So, one may expect 1:6 stoichiometry in the Cr-H system, by analogy with niobium and tungsten hydrides^11^,^20^. At ambient conditions, only the experimentally known 1:1 ratio was found, while at 160 GPa, numerous hydrogen-rich CrH_*n*_ phases with n up to 8 were identified. It is noteworthy that our evolutionary searches revealed that the 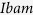-Cr_2_H_5_ structure was dynamically and thermodynamically stable when pressure is higher than 160 GPa (without ZPE correction, SM Fig. S1). However, when ZPEs are added, this 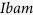 symmetry Cr_2_H_5_ structure is the most stable phase but it becomes unstable with respect to disproportionation reaction (+0.014 eV/atom above the convex hull at 300 GPa).

**Figure 2:**
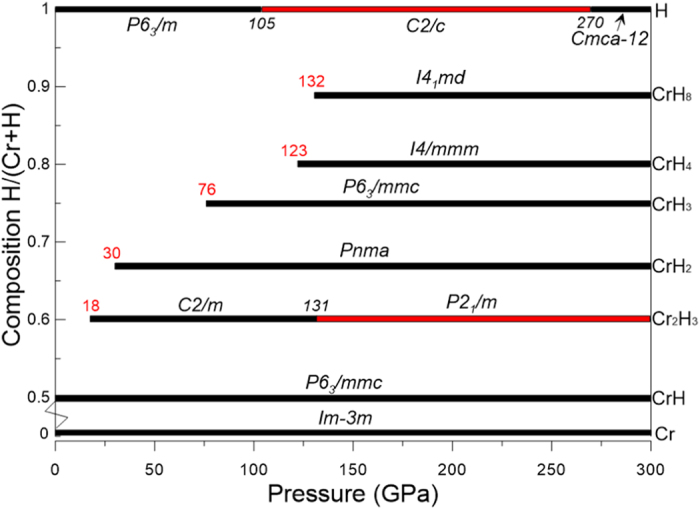
Pressure-composition phase diagram for the Cr-H system in the pressure range from 0 to 300 GPa.

A Cr_2_H_7_ phase with 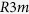 symmetry has the most negative enthalpy of formation of any of the 2:7 stoichiometric structures examined until 300 GPa and phonon calculations revealed that it was dynamically stable at 160 GPa. Its crystal structure is shown in SM Fig. S3. Nevertheless, 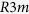-Cr_2_H_7_ did not fall on the convex hull (with and without ZPE corrections, see SM Tab. S2) and therefore it is unlikely that the Cr_2_H_7_ phase will be particularly stable with respect to decomposition into other chromium hydrides and/or solid H_2_ and chromium. The pressure-composition phase diagram (with ZPE correction) is shown in Fig. 2. Detailed structural information, enthalpies of formation, and phonon dispersion curves are presented in SM Tab. S3 and SM Fig. S4. Notably, structural searches revealed that no Cr-rich compounds are stable in the studied pressure range.

**Figure 3:**
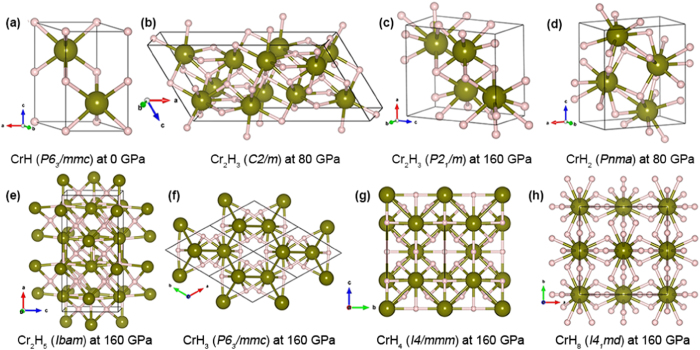
Crystal structures of the newly discovered Cr-H compounds. Large spheres represent chromium atoms, small spheres denote hydrogen atoms.

Crystal structures of the newly discovered Cr-H compounds for each stoichiometry are shown in Fig. 3. A common feature of the CrH, Cr_2_H_3_, CrH_2_ and Cr_2_H_5_ structures is that the metal frameworks effectively form hexagonal close-packed (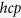) sublattice, in which hydrogen atoms occupy either the octahedral sites and/or the tetrahedral sites. In the anti-NiAs-type structure of CrH (Fig. 3a), hydrogen atoms occupy all octahedral voids. The calculated shortest Cr-Cr and Cr-H bonds are 2.394 Å and 1.695 Å at 160 GPa, respectively. This Cr-Cr distance is much longer than that in the pure chromium at the same pressure (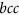, 2.211 Å). Its corresponding isoelectronic analogues MoH^10^ and WH^11^ also crystallize in this 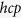 structure at low pressures. It is noteworthy that all the octahedral interstitial sites are filled, while the tetrahedral sites are vacant in CrH. Additional hydrogen atoms could only be inserted into the tetrahedral sites if the metal sublattice is not changed. The hydrogen-rich hydrides Cr_2_H_3_, CrH_2_ and Cr_2_H_5_ verify these expectations.

Cr_2_H_3_ becomes stable in the monoclinic 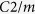 structure at pressures higher than 18 GPa, shown in Fig. 3(b). The metal 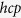 framework is slightly distorted with a quarter of the tetrahedral sites filled by hydrogen atoms, resulting in lowering of the symmetry. At 131 GPa, the 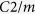 phase transforms to another monoclinic structure with space group 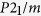, as shown in Fig. 3(c). Those two structures have similar topologies. One-third of the hydrogen atoms occupy the tetrahedral sites, while others occupy the octahedral sites. The minor differences arise from different hydrogen packing. This alternative hydrogen packing also results in different coordination environments of the central chromium atoms eventually yielding two distinct phases. At 160 GPa, the shortest Cr-Cr and Cr-H bonding lengths are 2.358 Å and 1.549 Å, respectively.

For CrH_2_, the ground-state structure adopts an orthorhombic 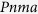 structure (Fig. 3d), and it becomes thermodynamically stable at pressures higher than 30 GPa. The metal 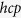 sublattice is squeezed along the *c* axis direction and half of the tetrahedral sites are filled by hydrogen atoms. At 160 GPa, the shortest Cr-Cr and Cr-H bond lengths are 2.399 Å and 1.551 Å, respectively. Compared with CrH and Cr_2_H_3_, we can find that the metal 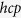 sublattice is only slightly changed, the extra hydrogen atoms are just inserted into the tetrahedral interstitial sites. Note that CrH_2_ has been reported to adopt the CaF_2_-type structure (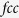 metal sublattice) at ambient pressure^21^; however, we found that the 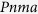 structure is enthalpically more favorable in the pressure range of 30 ∼ 300 GPa. This newly discovered 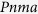 structure for CrH_2_ has also been proposed by Zaleski-Ejgierd *et al.*^11^ for its isoelectronic analogue WH_2_ above 50 GPa. In fact, the 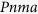 structure is adopted by most alkali earth dihydrides (MgH_2_, CaH_2_, SrH_2_ and BaH_2_) and WN_2_ above 34 GPa^22^.

Our evolutionary searches revealed that Cr_2_H_5_ crystallizes in the 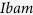 structure (Fig. 3e), however, it becomes metastable after inclusion of ZPE correction. In this structure, the metal 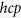 framework is severely distorted with the shortest Cr-Cr bond length of 2.486 Å at 160 GPa, much larger than the perfect 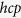 framework (2.394 Å) in CrH. Three quarters of the tetrahedral sites are filled by hydrogen atoms, while other hydrogen atoms are inserted into the octahedral voids. The shortest Cr-H bond length is 1.591 Å at 160 GPa. We conclude that the Cr_2_H_3_, CrH_2_ and Cr_2_H_5_ structures are versions of the perfect anti-NiAs-type CrH with ordered tetrahedral interstitial sites filled by hydrogen atoms.

We also found two host-guest structures, CrH_3_ and CrH_4_. CrH_3_ emerges on the phase diagram at 76 GPa and adopts a 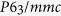 structure (Fig. 3f). This 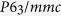 structure consists of metal honeycomb framework in which each hydrogen acts as a bridging atom shared by four chromium atoms. Each chromium atom is coordinated by 12 hydrogen atoms at the same distance. At 160 GPa, the shortest Cr-H bond length is 1.670 Å, while the second-nearest Cr-H distance is 1.759 Å. The preferred structure found for CrH_4_ has space group 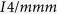 and is shown in Fig. 3(g). CrH_4_ is thermodynamically stable at pressures above 123 GPa and at least to 300 GPa. In this structure, the metal sublattice has a relatively complex topology, with each chromium atom now coordinated by 14 hydrogen atoms. The shortest Cr-H distance is 1.656 Å at 160 GPa, much longer than in CrH_2_ or CrH_3_. This structure also exhibits features typical of a host-guest structure, where hydrogen atoms occupy the cage cavities formed by metal atoms. Note that the 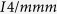 symmetry has also been proposed for IrH_4_, however, IrH_4_ is thermodynamically unstable with respect to disproportionation into hydrogen and lower hydrides^3^.

One can see that no H_2_ pairs (which we define by condition 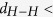
1.4 Å) are found in Cr_2_H*n* (n 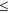 8). At all pressures at least up to 300 GPa, all H-H separations are longer than 1.4 Å in our ground-state phases. Since hydrogen remains a molecular solid up to ∼500 GPa^23^, and atomic packing in molecular crystals is not dense, we can expect increasingly H-rich chromium hydrides with increasing pressure. Indeed, we discovered a 1:8 stoichiometry with space group 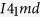 (Fig. 3h), and the phase diagram shows that it is thermodynamically stable at pressures above 132 GPa and at least to 300 GPa. We note that, in contrast to chromium, niobium and tungsten hydrides present stable high-pressure phases with a 1:6 stoichiometry as proposed from theoretical calculations^11^,^20^.

### Bonding in stable Cr-H compounds

**Figure 4:**
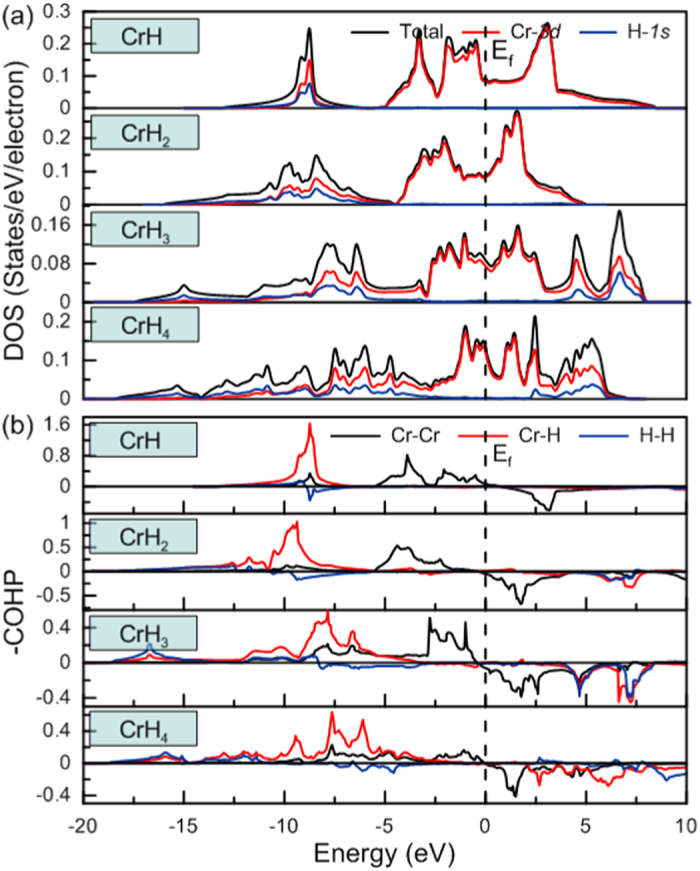
The calculated electronic properties of CrH_*n*_ (n = 1–4) phases at 160 GPa. (**a**) Total and projected densities of states (per valence electron, DOS); (**b**) Crystal orbital Hamilton population (COHP). The dashed line denotes the Fermi level (E_*f*_).

To examine chemical bonding, we calculated the total and projected densities of states (DOS). We focus here on the characterization of the four stable stoichiometries, CrH_*n*_ (n = 1–4) at 160 GPa, and the results are shown in Fig. 4(a). The DOS of the CrH_*n*_ compounds mainly decompose into two well-separated energy regions: (1) a hybridized Cr-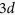

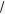
H-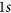 band, 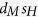, and (2) a partially-filled higher-lying Cr-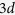 band, 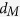. The 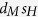 band is the result of strong hybridization between the 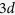 level of Cr atoms and the 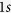 level of H atoms. The 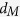 band is dominated by 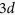 orbitals of Cr atoms and is responsible for metallicity. These results confirm the mixed covalent and metallic characteristics of CrH_*n*_ (n = 1–4) compounds. Additionally, the DOS indicates a clear depletion of density of states close to/at the Fermi level in a deep pseudogap, indicating that the four hydrides should be considered as weak metals. When n increases from 1 to 4 in the CrH_*n*_ series, the width of the 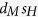 band expands. This energy dispersion is caused by more extensive mixing between Cr-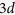 and H-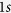 orbitals, and suggests enhancement of covalency of the Cr-H bond network.

**Figure 5:**
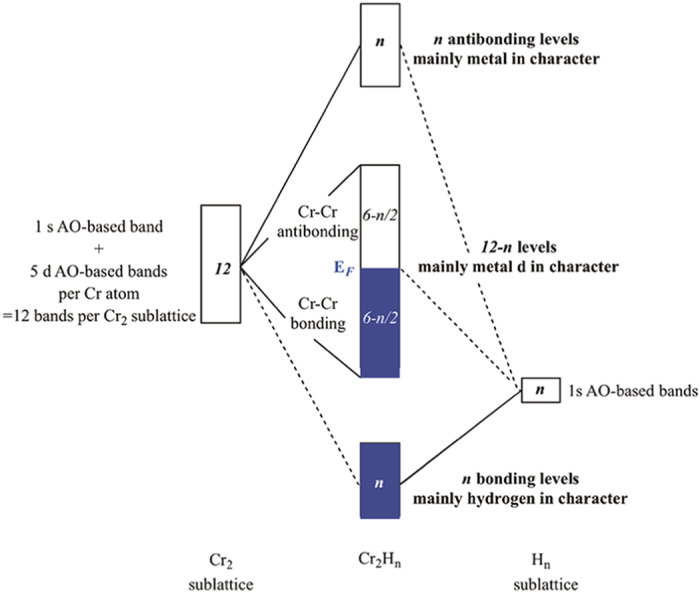
Schematic interaction diagram of chromium and hydrogen sublattices to form Cr_2_H_*n*_ (n = 2–8) phases. The Fermi level is at the top of the bonding metal-metal *d*-block. Blue boxes indicate occupied levels, open ones are vacant.

Interatomic interactions were further explored using the crystal orbital Hamilton populations (COHP) and integrated crystal orbital Hamilton populations (ICOHP)^24^, computed using the linear muffin-tin orbital (LMTO) method^25^, and the results are shown in Fig. 4(b) and SM Fig. S5. The ICOHP value tends to scale with bond strength in compounds. One can see that Cr-Cr bonding peaks exist up to the Fermi level. To explain this peculiar signature, we used the orbital approach to analyze the electronic structures of Cr_2_H_*n*_ (n = 2–8), which was displayed in Fig. 5.

If we consider only the five 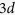 and one 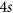 orbitals of Cr (thus 6 atomic orbitals, 6 AO), the chromium sublattice in Cr_2_H_*n*_ has then 12 atomic orbitals-based crystal orbitals, i.e. 12 bands. They are schematized by a box at the left part of the orbital interaction diagram in Fig. 5. Going now to the hydrogen sublatice where long H-H contact exist (d_*H*−*H*_ 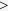 1.4 Å, small *s*-*s* orbital overlap), one may figure out that n hydrogen atoms have n molecular orbitals, thus n localized bands as depicted in the right of Fig. 5. H is more electronegative than Cr, so these localized H levels are lower in energy than the Cr ones. H_*n*_ and Cr_2_ sublattices interact, up to (12 + n) levels are allowed to mix, if symmetry conditions are encountered; n levels are stabilized in energy and are Cr-H bonding, mainly H character. Thus, on a total of (12 + n) levels, n bands are destabilized due to the metal-ligand out-of-phase antibonding overlap combinations. These are mainly metal in character, and are vacant. Thus, on a total of (12 + n) levels, (12-n) levels remain at the same energy region at a first glance and have Cr-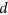 character. These atomic orbitals-based bands are split in (6-n/2) bonding and (6-n/2) antibonding levels, forming the so-called metal 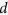 bands region.

The bottom of the 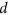-band region should be metal-metal bonding and the top metal-metal antibonding. Note that, for clarity, we have chosen to separate metal-ligand bonding, metal 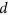-bands and metal-ligand antibonding energy regions sketched by three separated boxes, even though these bands overlap (see computed DOS in Fig. 4). Cr_2_H_*n*_ has (12 + n) valence electrons also, in a low-spin configuration, n bonding levels are filled by 2n electrons (H^−^ hydridic levels). Thus (12-n) electrons (12
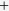
n
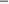
2n) remain on (12-n) Cr orbitals. Thus half on this 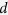-block is occupied for Cr_2_H_*n*_ and then the Fermi level is at the top of the bonding Cr-Cr peak. Our expectation is confirmed by the calculated COHP curves in Fig. 4 where the Cr-Cr bonding and antibonding features are clearly seen. This simple model based on molecular orbital concept gives us some clear insights into the thermodynamic stability of the proposed high-pressure Cr_2_H_*n*_ phases. Looking at the DOS, we see that bonding M-H regions expand with increasing H content. An occupied band of greater dispersion is connected to the enhancement of the covalent character of the metal-ligand interaction. Analysis of the ICOHP (SM Fig. S5) provides additional evidence for our conclusion: Cr-H values increase with increasing H content, indicating that Cr-H bonds become stronger, while Cr-Cr bonds weaken.

**Figure 6:**
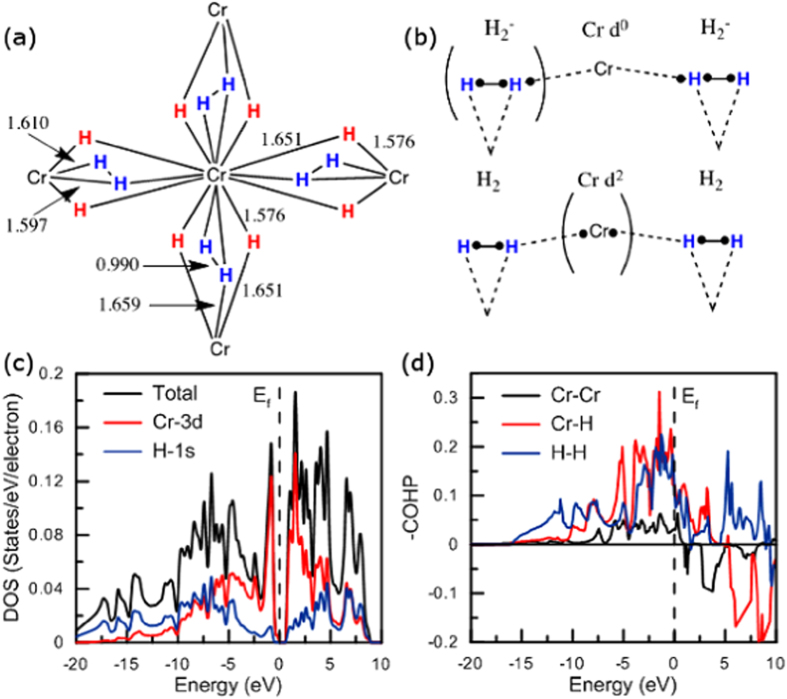
(**a**) Schematic local coordination for CrH_8_. H_2_ units are in blue while hydridic H^−^ ligands are in red; (**b**) Cr-*η*^1^-H_2_ bonding: two resonant Lewis structures; (**c,d**) DOS and COHP for CrH_8_ at 160 GPa.

In CrH_8_, if one considers a total valence electron transfer from the electropositive element (6e^−^ of Cr) to the anionic network, two extra electrons are needed to get formally 8 hydridic H^−^. Therefore, covalent bond between hydrogen atoms may form as a requisite to this electron count situation. This is what happens in 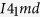 CrH_8_ phase. Effectively, H_2_ pairs are found with a H-H distance of 0.990 Å at 160 GPa, much longer than that in free H_2_ (0.74 Å), but similar to that in CsH_3_ (0.95 Å in linear 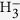). Each H_2_ unit binds side-on (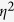-H_2_) to metal at a Cr-H distance of roughly 1.61 Å. A peculiar bonding mode is also observed in these 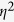-H_2_ ligands: they are bonded through one hydrogen to one adjacent chromium atom at only 1.659 Å at 160 GPa. Finally, eight hydrogen ligands are in a bridging position (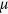-H) with Cr-(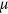-H) bond lengths of 1.58–1.65 Å. To summarize, each chromium center is linked to 14 hydrogen atoms as depicted in Fig. 6(a).

While Cr_2_H_*n*_ (n = 1–8) are metallic, CrH_8_ is a semiconductor (DOS, Fig. 5c). This finding may be well explained if one considers the following Zintl picture: the four hydride H^−^ ligands per chromium follow the duet rule, anionic 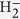 ligand is a three-electron donor with a singly occupied 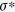
(H_2_), which may explain the elongated H-H bond found in H_2_ units (0.990 Å at 160 GPa), thus Cr has a +6 formal oxidation state, i.e. is a 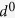 species. Obviously, another way to assign valence electrons could be advocated (Fig. 6b). Let us analyze the bonding mode encountered in CrH_8_. First, the bridging hydride bonding mode, Cr-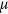-H-Cr, is well known: this interaction can be described by a 3 center-2 electron (3c-2e) bonding scheme, which renders well the observed slightly Cr-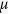-H elongation (1.58–1.64 Å at 160 GPa). The occupied 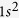 orbital of H^−^ interacts with the appropriate vacant metal orbitals of the Cr_2_ fragment, to form an occupied stabilized bonding molecular orbital (MO) and a vacant one. Next, the Cr-(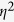-H_2_) bonding scheme may be viewed also as a classical 3c-2e bonding scheme as found in molecular triangular 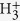 or Kubas-type complexes such as Cr(CO)_5_(H_2_)^26^.

Finally, let us focus on the short Cr-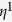-H_2_ bond computed at 1.659 Å at 160 GPa. As each metal center interacts with one hydrogen atom of the 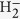 ligand (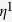-coordination mode), a delocalized 3c-3e bonding scheme may be invoked, as depicted in Fig. 6(b). The bonding may be described by the following interactions: a delocalized occupied MO can be formed from a 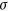 orbital of H_2_, and the appropriate vacant 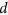 orbital of the metal atom (agostic bond)^27^, but also a stabilized MO can be formed from the singly occupied 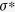
(H_2_) and a vacant *d* orbital. Detailed analysis shows that the chromium *d* states contribute to the DOS near the Fermi level and a strong positive metal-hydrogen overlap occurs in this energy region, signature of a bonding situation. All of these deficient bonding schemes explain well the elongated H-H, Cr-(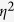-H_2_) and Cr-(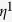-H_2_) bonds encountered in the Cr-(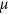-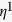, 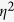-H_2_)-Cr fragment and in bridging hydrides. We should recall that paired hydrogen atoms are found in the thermodynamically and mechanically stable (>132 GPa) 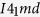 CrH_8_, not in the isoelectronic solid state WH_8_ compound^11^.

### Superconductivity of CrH and CrH_3_

**Figure 7:**
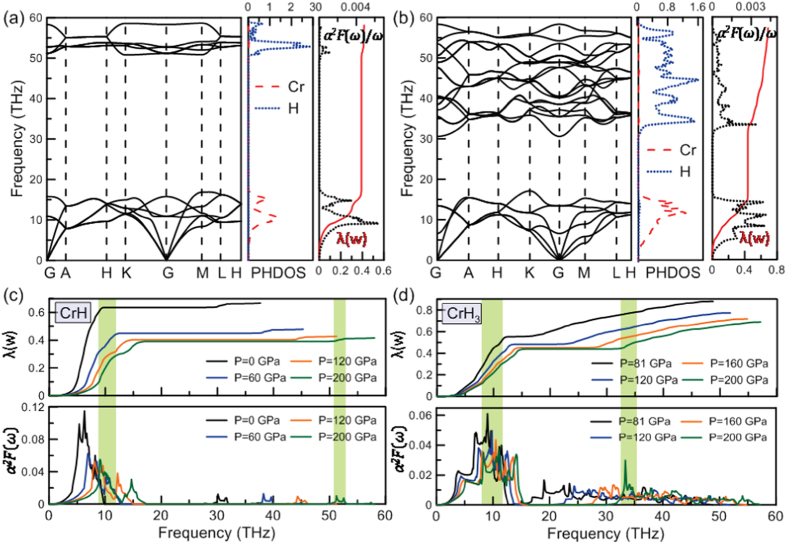
(**a,b**) show phonon dispersions, partial phonon density of states (PHDOS), spectral functions 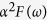/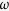 and electron-phonon coupling strength *λ*(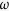) for CrH and CrH_3_ at 200 GPa, respectively; (**c,d**) represent 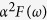 and *λ*(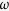) at selected pressures. Shaded regions show the significant contribution of two strong peaks of 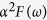 to *λ*.

Recently, it has been suggested that hydrogen-rich compounds might present high-temperature superconductivity^28^. A series of hydrogen-rich compounds have been extensively explored because their metallization can occur at lower pressures^29^,^30^,^31^,^32^,^33^,^34^,^35^,^36^. Here, we explored superconductivity of CrH and CrH_3_ - as representative Cr_2_H_*n*_ (n = 2–8) phases - by performing electron-phonon coupling (EPC) calculations. The phonon dispersions, partial phonon density of states (PHDOS), Eliashberg spectral functions 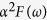 and electron-phonon coupling integration of *λ*(*ω*) for CrH and CrH_3_ at 200 GPa were explicitly calculated to explore the potential superconductivity of CrH and CrH_3_ (Fig. 7).

**Figure 8:**
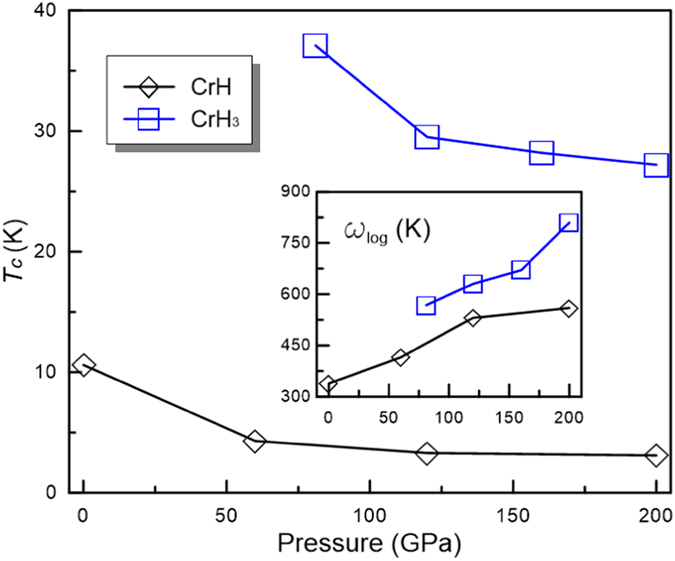
Pressure dependence of *T*_*c*_ for CrH and CrH_3_ phases. Inset shows the logarithmic average phonon frequency (*ω*_*log*_) with pressure.

For the anti-NiAs-type CrH, bands below 18 THz are mainly due to chromium vibrations, while higher-frequency modes (in the range 50 ∼ 60 THz) are mostly related to the hydrogen atoms. For CrH_3_, the low-frequency vibrations are similar to the case of CrH, while the high-frequency modes are spread over the range of 30 ∼ 60 THz. The critical temperature of superconductivity 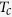 was estimated by using the Allen-Dynes equation^37^ with a typical choice of Coulomb pseudopotential μ^*^ = 0.1 and 0.13. It is found that the anti-NiAs-type CrH has a high potential to be superconductive at atmospheric pressure. The calculated *λ* is 0.67 at 0 GPa and 0.42 at 200 GPa. Using the calculated logarithmic average frequency (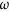
_*log*_) of 338.5 K and commonly accepted μ^*^ = 0.1, the estimated 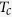 is 10.6 K at 0 GPa. To the best of our knowledge, this is the first identification of a superconductive metal hydride at ambient pressure. In addition, we found that pressure has a negative effect on 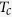 (Fig. 8). The calculated 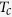 decreases monotonically with pressure from 10.6 K at 0 GPa to 3.1 K at 200 GPa. From Fig. 7(a), we found that coupling of the electrons with chromium vibrations in the frequency region below 18 THz contributes ∼93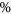 of the total *λ*, while the remaining 7% is from hydrogen vibrations (50 ∼ 60 THz). Our calculations also show that CrH will be less stable than the mixture of CrH_0.97_^12^ and H_2_ at temperatures above 1337 K (SM Fig. S8), but more stable at temperatures below 1337 K. This explains why high-temperature synthesis produces slightly off-stoichiometric materials (e.g. CrH_0.97_) and confirms that stoichiometric superconducting CrH is indeed a ground state at lower temperatures.

For CrH_3_, the EPC constant *λ* was calculated to be 0.95 at 81 GPa and 0.69 at 200 GPa, which is much larger than that of CrH and indicative of a much stronger EPC interaction. The calculated 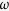
_*log*_ is 568.1 K at 81 GPa. With these numbers, the estimated 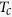 is 37.1 K (μ* = 0.1), comparable to the extensively studied MgB_2_ (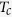 = 39 K^38^). Furthermore, the strong coupling of electrons with chromium vibrations, which dominate the low-frequency phonon spectra, contributes ∼64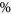 of the total *λ*, while coupling of electrons with hydrogen vibrations contributes 36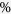 of *λ*, which play a more significant role than in CrH. The values of 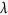, 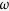
_*log*_ and 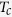 for the CrH and CrH_3_ phases at selected pressures are shown in SM Tab. S4.

We found that pressure has a negative effect on *λ* for both CrH and CrH_3_ phases, indicating the electron-phonon coupling will become weaker on increasing pressure (SM Fig. S9). 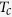 for both structures will decrease as pressure increases (Fig. 8). The same were found for 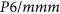-BH^39^ and 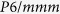-MgB_2_ structures^40^. Our calculations reveal that DOS at the Fermi level is mainly Cr-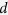 in character and decreases as pressure increases (SM Fig. S10). For both materials, pressure leads to the decrease of 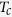. Additionally, superconductivity enhances with increasing hydrogen content, indicating increasing role of the hydrogen sublattice (“metallic hydrogen”) in determining superconductivity.

## Conclusion

Systematic search for stable compounds in the Cr-H system at pressures up to 300 GPa revealed eight new compounds Cr_2_H_*n*_ (n = 2–8, 16), and all the predicted structures are dynamically stable. For Cr_2_H_*n*_ (n = 2–5), when the amount of hydrogen increases, firstly the octahedral sites of the metal 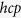 sublattice are filled (see CrH), followed by filling at the tetrahedral voids (i.e. Cr_2_H_5_). Further addition of hydrogen atoms in Cr_2_H_*n*_ (n > 5) will lead to a reorganization of the chromium sublattice. Two host-guest structures (CrH_3_ and CrH_4_) are predicted where hydrogen atoms fill channels of the Cr sublattice. Additionally, CrH_8_ has the highest hydrogen concentration and H_2_ units are identified in this structure.

Our analyses of the electronic structure and orbital interactions revealed the bonding nature of high-pressure chromium hydrides. Chromium was found to form short, primarily covalent bonds with hydrogen. Electron-phonon coupling calculations showed that the CrH and CrH_3_ phases are phonon-mediated superconductors. Our study unravels a superconductive potential of CrH at atmospheric pressure with 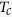 of 10.6 K at 0 GPa. To the best of our knowledge, this is the first identification of a superconductive metal hydride at ambient pressure. The superconductivity in CrH_3_ is enhanced by the metallic hydrogen sublattice with 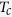 of 37.1 K at 81 GPa. Superconductivity of CrH and CrH_3_ comes largely from strong coupling of the electrons with Cr vibrations, and coupling with H vibrations becomes more important with increasing H content.

## Methods

To search for stable Cr-H compounds with variable compositions under high pressure, we employed an effective and unbiased crystal structure searching method based on an evolutionary algorithm, as implemented in the USPEX code^41^,^42^,^43^,^44^. All compositions between pure Cr and pure H were allowed under constraint that the total number of atoms in the unit cell be up to 30. The first generation of 100 structures/compositions was produced randomly; all subsequent generations contained 80 structures and were produced using variation operators such as heredity (60%), softmutation (15%), transmutation (15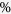), 10% of each new generation was produced randomly.

Geometry relaxations were performed based on density functional theory (DFT)^45^,^46^ with the Perdew-Burke-Ernzerhof (PBE)^47^ version of the generalized gradient approximation functional (GGA), as implemented in the VASP code^48^. We used projector-augmented wave (PAW)^49^ potentials for Cr and H atoms, with radii 2.5 a.u. for Cr ([Ar] core) and 1.1 a.u. for H. A plane-wave basis set with a kinetic energy cutoff of 600 eV was employed. We used uniform 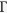-centered *k*-points meshes with a reciprocal space resolution of 2*π* × 0.03 Å^−1^ for Brillouin zone sampling. All structural relaxations were done with a tight force convergence threshold (1 meV/Å). These settings enable excellent convergence of total energy differences, forces, and stress tensors.

Electron-phonon coupling (EPC) calculations were performed using the Quantum 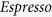 package^50^ with Vanderbilt-type ultrasoft pseudopotentials^51^ with cutoff energies of 60 and 500 Ry for the wave functions and the charge density, respectively. The dynamical matrix was computed on an 8 × 8 × 5 mesh for CrH and 4 × 4 × 5 mesh for CrH_3_. In all calculations, force convergence threshold (1 meV/Å) for structural optimizations was applied. The McMillan-Allen-Dynes equation^37^ was used to estimate *T*_*c*_, as follows: 
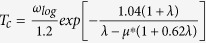
, where *ω*_*log*_ is the logarithmic average frequency, *λ* is the electron-phonon coupling constant and μ* is the Coulomb pseudopotential, which is taken to be between 0.1–0.13^28^.

Theoretical phonon spectra were calculated based on the supercell approach using the PHONOPY package^52^ in order to probe the dynamical stability of the enthalpically preferred Cr_*x*_H_*y*_ compounds at different pressures. Because of the very low mass of the hydrogen atom, the zero-point energy (ZPE) may well be large enough to affect the overall structural stability of the computed phases. Therefore, we estimated the ZPEs for each Cr_*x*_H_*y*_ compound, pure H_2_ and Cr under pressure within the quasi-harmonic approximation using the PHONOPY code. The results are summarized in Tab. S2 in Supplementary Materials (SM). All given energies are ZPE corrected. Note that the ZPE contributions to the enthalpies altered the transition phase pressures and stability range pressures by roughly 10%, but they did not affect the identification of the ground-state phases, except that Cr_2_H_5_ becomes metastable when ZPE is taken into account.

The enthalpy of formation per atom was calculated as: 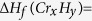

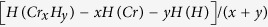
. After verifying the dynamical stability of the Cr-H compounds via phonon calculations, we constructed convex hull diagrams for the Cr-H system at different pressures. At a given pressure, compounds located on the convex hull are stable against decomposition into other compositions, whereas compounds above the convex hull are metastable and will decompose into the compounds located on the convex hull. Any structure for which the enthalpy of formation lies on the convex hull is by definition thermodynamically stable and - in principle - can be synthesized experimentally^53^,^54^.

## Additional Information

**How to cite this article**: Yu, S. *et al.* Pressure-driven formation and stabilization of superconductive chromium hydrides. *Sci. Rep.* **5**, 17764; doi: 10.1038/srep17764 (2015).

## Supplementary Material

Supplementary Information
