# Supplementary material for: Pressure-driven formation and stabilization of superconductive chromium hydrides
Source: Sci Rep. 2015 Dec 2;5:17764. doi: 10.1038/srep17764 (PMC4667211; doi:10.1038/srep17764)
Supplement: Supplementary Information [file srep17764-s1.pdf]

Pressure-driven formation and stabilization of superconductive chromium hydrides

Supplementary Materials

Shuyin Yu[1,2,*], Xiaojing Jia[1], Gilles Frapper[3], Duan Li[1,2], Artem R. Oganov[4,5,6,2], Qingfeng Zeng[1,2], and Litong Zhang[1]

[1] *Science and Technology on Thermostructural Composite Materials Laboratory, School of Materials Science and Engineering, Northwestern Polytechnical University, Xi'an, Shaanxi 710072, PR China*
[2] *International Center for Materials Discovery, School of Materials Science and Engineering, Northwestern Polytechnical University, Xi'an, Shaanxi 710072, PR China*
[3] *IC2MP UMR 7285, Université de Poitiers - CNRS, 4, rue Michel Brunet TSA 51106 - 86073 Poitiers Cedex 9, France*
[4] *Skolkovo Institute of Science and Technology, 5 Nobel Street, Skolkovo 143025, Russia*
[5] *Department of Geosciences, Center for Materials by Design, and Institute for Advanced Computational Science, State University of New York, Stony Brook, NY 11794-2100, USA*
[6] *Moscow Institute of Physics and Technology, Dolgoprudny, Moscow Region 141700, Russia*

[*] Correspondence and requests for materials should be addressed to Shuyin Yu (yushuyin2014@gmail.com)

The supplementary materials contains:

- Convex hull diagrams of Cr-H system with stable and metastable structures at selected pressures (**Fig. S1**)
- The relative stability of CrH with and without considering magnetism (**Tab. S1**)
- Pressure-composition phase diagram for the Cr-H system without zero-point energy correction (**Fig. S2**)
- Crystal structure of the metastable Cr_2H_7 phase (**Fig. S3**)
- The enthalpies, zero-point energies (ZPE) and ZPE-corrected enthalpies of the Cr-H compounds at selected pressures (**Tab. S2**)
- The structural parameters of the predicted structures (**Tab. S3**)
- Phonon spectra for Cr_xH_y compounds at selected pressures (**Fig. S4**)
- The integrated crystal orbital Hamilton population at 160 GPa (**Fig. S5**)
- Electron localization function distributions for stable CrH_n (n=1-4) at 200 GPa (**Fig. S6**)
- The calculated entropy of H_2 gas at different temperatures (**Fig. S7**)
- The temperature dependence of free energy of the suggested chemical reaction $Cr_{36}H_{35}$ (s) + 0.5 H_2 (g) = 36 CrH (s) (**Fig. S8**)
- The calculated λ, ω_{log} and T_c values for CrH and CrH_3 using $\mu^* = 0.1$ and 0.13 at selected pressures (**Tab. S4**)
- The EPC parameter λ as a function of pressure for CrH and CrH_3 (**Fig. S9**)
- The total electronic densities of states around Fermi level for CrH and CrH_3 at selected pressures (**Fig. S10**)
- Predicted ground-state stable structures at (a) Cr_2H_5 (*Ibam* at 160 GPa), (b) CrH_3 (*$P6_3/mmc$* at 160 GPa) and (c) CrH_4 (*I4/mmm* at 160 GPa) (**Fig. S11**)

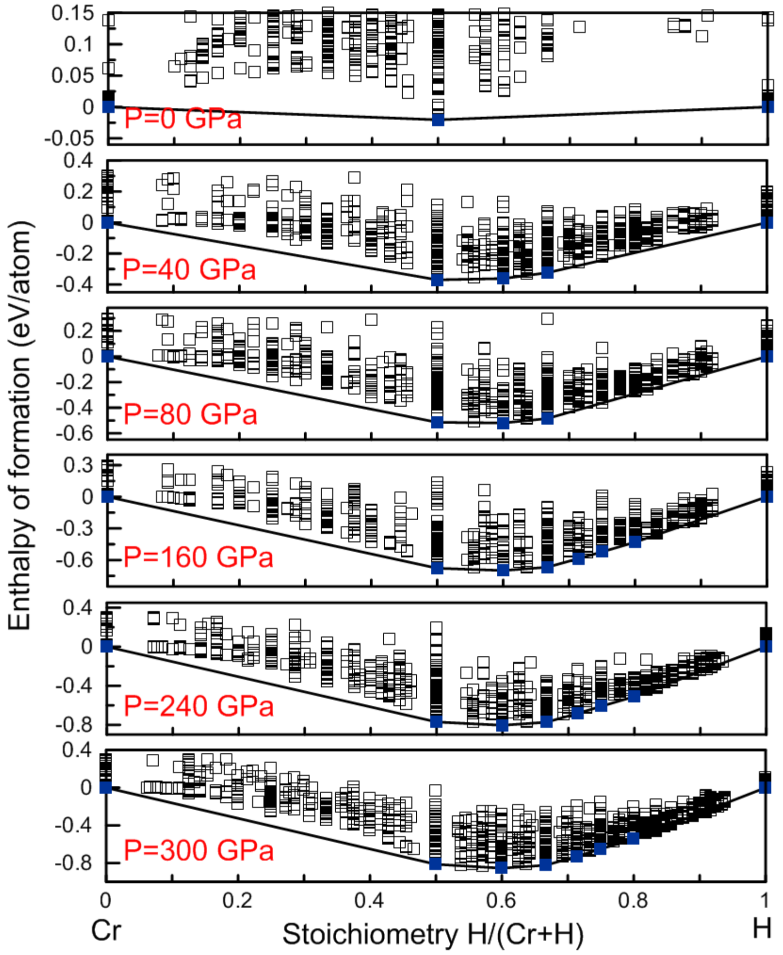

Fig. S1 (color online) Convex hull diagrams of Cr-H system at 0, 40, 80, 160, 240 and 300 GPa. The solid blue squares denote the stable structures, black squares represent metastable structures.

Tab. S1 Effect of magnetism correction on the relative stability of Cr (*Im-3m*) and CrH (*P6₃/mmc*) at 0 GPa and 40 GPa. Energies and formation enthalpies are given in eV/atom.

**Energy**	**Magnetism correction***	**without magnetism correction**
$Cr_{(s)}$ at 0 GPa	-9.518	-9.510
$Cr_{(s)}$ at 40 GPa	-6.829	-6.831
$CrH_{(s)}$ at 0 GPa	-6.467	-6.467
$CrH_{(s)}$ at 40 GPa	-4.898	-4.898
**Formation Enthalpy**	**CrH = Cr + ½ H_2**	
0 GPa	-0.016	-0.020
40 GPa	-0.375	-0.374

*Cr was treated as antiferromagnetic, all other phases are non-magnetic in the ground state.

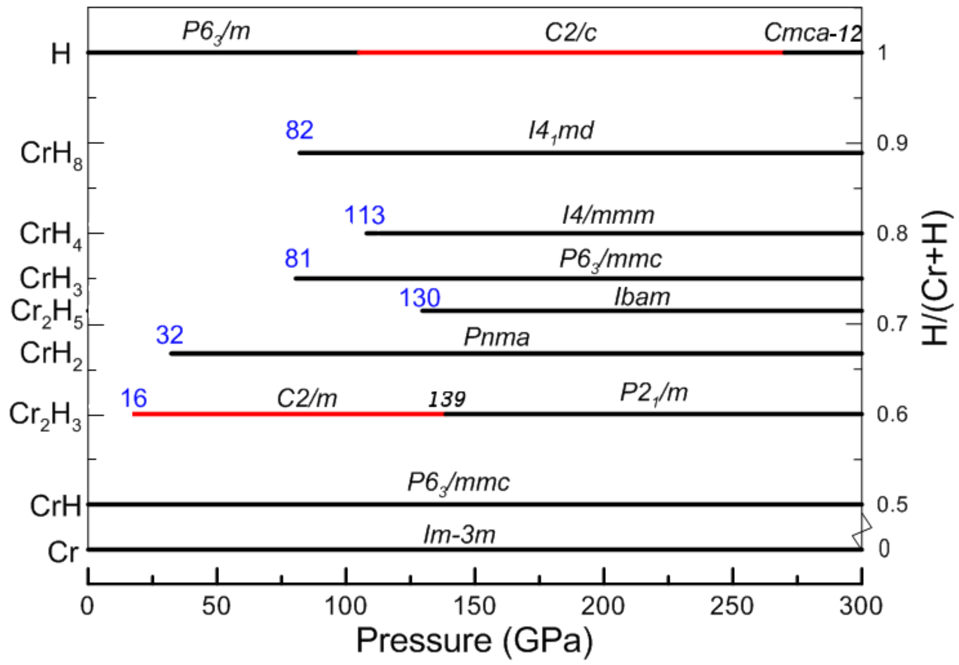

Fig. S2 (Color online) Pressure-composition phase diagram for the Cr-H system in the pressure range from 0 to 300 GPa without zero-point energy correction. The blue pressure values indicate the initial pressure at which the compound becomes unstable with respect to disproportionation into other chromium hydrides and/or chromium and hydrogen (dashed lines). The phase transition pressure is indicated in italics. Pressures are given in GPa.

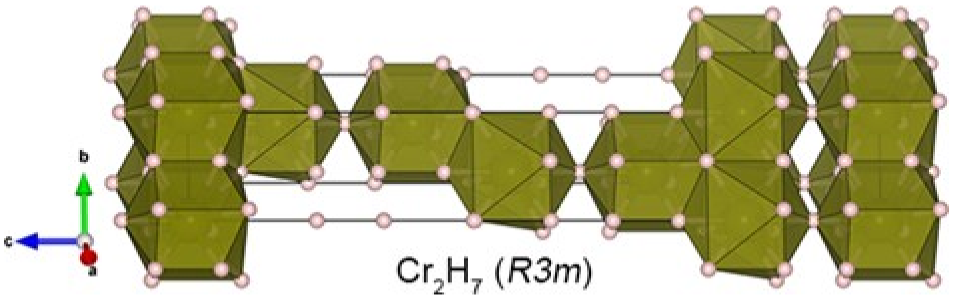

Fig. S3 (color online) Crystal structure of the metastable Cr_2H_7 phase. Large atoms denote chromium atoms, small atoms represent hydrogen atoms.

Tab. S2 The enthalpies, zero-point energies (ZPE) and ZPE-corrected enthalpies of the Cr-H compounds at selected pressures (energies in eV/atom).

**Phase**	**Space group**	**Pressure (GPa)**	**Enthalpy**	**ZPE**	**Enthalpy (ZPE-corrected)**
Cr	*Im-3m*	0	-9.510	0.045	-9.465
		40	-6.831	0.055	-6.776
		80	-4.415	0.060	-4.356
		160	-0.030	0.069	0.039
		240	3.946	0.075	4.021
		300	6.727	0.078	6.805
CrH	*$P6_3/mmc$*	0	-6.467	0.122	-6.346
		40	-4.898	0.148	-4.750
		80	-3.477	0.166	-3.311
		160	-0.909	0.193	-0.717
		240	1.418	0.212	1.630
		300	3.050	0.224	3.274
CrH	*Fm-3m*	0	-6.424	0.116	-6.308
		40	-4.849	0.143	-4.707
Cr_2H_3	*C2/m*	0	-5.800	0.144	-5.657
		40	-4.426	0.171	-4.255
		80	-3.192	0.191	-3.001
		160	-0.970	0.219	-0.751
		240	1.036	0.242	1.278
Cr_2H_3	*$P2_1/m$*	80	-3.190	0.191	-3.000
		160	-0.971	0.219	-0.752
		240	1.033	0.241	1.273
		300	2.436	0.254	2.690
CrH_2	*Pnma*	0	-5.330	0.155	-5.175
		40	-4.079	0.182	-3.897
		80	-2.964	0.202	-2.762
		160	-0.967	0.232	-0.735
		240	0.830	0.254	1.084
		300	2.086	0.268	2.354
Cr_2H_5	*Ibam*	0	-4.969	0.160	-4.809
		40	-3.793	0.187	-3.606
		80	-2.754	0.209	-2.545
		160	-0.904	0.238	-0.666
		240	0.754	0.260	1.013
		300	1.910	0.273	2.182
CrH_3	*$P6_3/mmc$*	0	-4.711	0.129	-4.582
		40	-3.586	0.161	-3.426
		80	-2.600	0.184	-2.416

		160	-0.855	0.215	-0.640
		240	0.700	0.236	0.937
		300	1.782	0.247	2.029
Cr_2H_7	*R3m*	0	-4.472	0.147	-4.325
		40	-3.383	0.178	-3.205
		80	-2.434	0.200	-2.234
		160	-0.761	0.227	-0.534
		240	0.727	0.248	0.975
		300	1.761	0.260	2.021
CrH_4	*I4/mmm*	0	-4.314	0.130	-4.183
		40	-3.267	0.159	-3.108
		80	-2.368	0.187	-2.181
		160	-0.783	0.220	-0.563
		240	0.624	0.243	0.867
		300	1.601	0.257	1.857
CrH_8	$I4_1md$	0	-3.774	0.179	-3.595
		40	-2.770	0.200	-2.570
		80	-1.985	0.219	-1.765
		160	-0.654	0.245	-0.409
		240	0.504	0.266	0.770
		300	1.299	0.279	1.578
H_2	$P6_3/m$	0	-3.284	0.148	-3.136
	(*0-105*)	40	-2.327	0.186	-2.141
	C2/c	80	-1.612	0.247	-1.365
	(*105-270*)	160	-0.434	0.281	-0.152
	Cmca-12	240	0.432	0.300	0.732
	(*270-300*)	300	1.001	0.311	1.312

Tab. S3 Calculated structural parameters of the predicted stable structures for Cr_xH_y compounds at selected pressures.

**Phase**	**Space group**	**Pressure (GPa)**	**Lattice parameters (Å)**	**Atomic coordinates (fractional)**		**H_f (ev/atom)**
CrH	$P6_3/mmc$	0	a=2.673	Cr (2c)	(0.667, 0.333, 0.75)	-0.020
			c=4.339	H (2a)	(0, 0, 0)	
Cr_2H_3	*C2/m*	80	a=9.242	Cr (4i)	(0.116, 0, 0.730)	-0.519
			b=2.580	Cr (4i)	(0.122, 0.5, 0.205)	
			c=4.517	H (2c)	(0, 1, 0.5)	
			β=61.7	H (2b)	(0, 0.5, 0)	

				H (4i)	(0.181, 0.5, 0.486)	
				H (4i)	(0.250, 0.5, 0.798)	
CrH_2	*Pnma*	80	a=4.161<br>b=2.639<br>c=4.670	Cr (4c)	(0.262, 0.75, 0.588)	-0.485
				H (4c)	(0.480, 0.75, 0.289)	
				H (4c)	(0.134, 0.25, 0.421)	
Cr_2H_3	$P2_1/m$	160	a=3.909<br>b=2.481<br>c=4.328<br>β=90.8	Cr (2e)	(0.744, 0.75, 0.574)	-0.699
				Cr (2e)	(0.268, 0.75, 0.903)	
				H (2e)	(0.999, 0.25, 0.714)	
				H (2e)	(0.514, 0.25, 0.744)	
				H (2e)	(0.137, 0.25, 0.083)	
Cr_2H_5	*Ibam*	160	a=7.354<br>b=3.727<br>c=3.558	Cr (8j)	(0.121, 0.767, 1)	-0.586
				H (8j)	(0.098, 0.193, 1)	
				H (8f)	(0.239, 0.5, 0.75)	
				H (4b)	(0.5, 1, 0.75)	
CrH_3	$P6_3/mmc$	160	a=3.510<br>c=2.456	Cr (2c)	(0.667, 0.333, 0.75)	-0.522
				H (6h)	(0.853, 0.147, 0.25)	
Cr_2H_7	*R3m*	160	a=2.491<br>c=15.750	Cr (3a)	(0, 0, 0)	-0.761
				Cr (3a)	(0, 0, 0.197)	
				H (3a)	(0, 0, 0.098)	
				H (3a)	(0, 0, 0.388)	
				H (3a)	(0, 0, 0.478)	
				H (3a)	(0, 0, 0.718)	
				H (3a)	(0, 0, 0.588)	
				H (3a)	(0, 0, 0.815)	
				H (3a)	(0, 0, 0.292)	
CrH_4	*I4/mmm*	160	a=3.564<br>b=7.010	Cr (4d)	(0, 0.5, 0.25)	-0.431
				Cr (2a)	(0, 0, 0)	
				H (16m)	(0.294, 0.294, 0.105)	
				H (4c)	(0.5, 0, 0)	
				H (4e)	(0, 0, 0.242)	
CrH_8	$I4_1md$	160	a=4.775<br>c=3.883	Cr (4a)	(0.5, 0.5, 0.946)	-0.265
				H (8b)	(0.296, 0.5, 0.272)	
				H (8b)	(0, 0.823, 0.814)	
				H (16c)	(0.187, 0.145,0.164)	

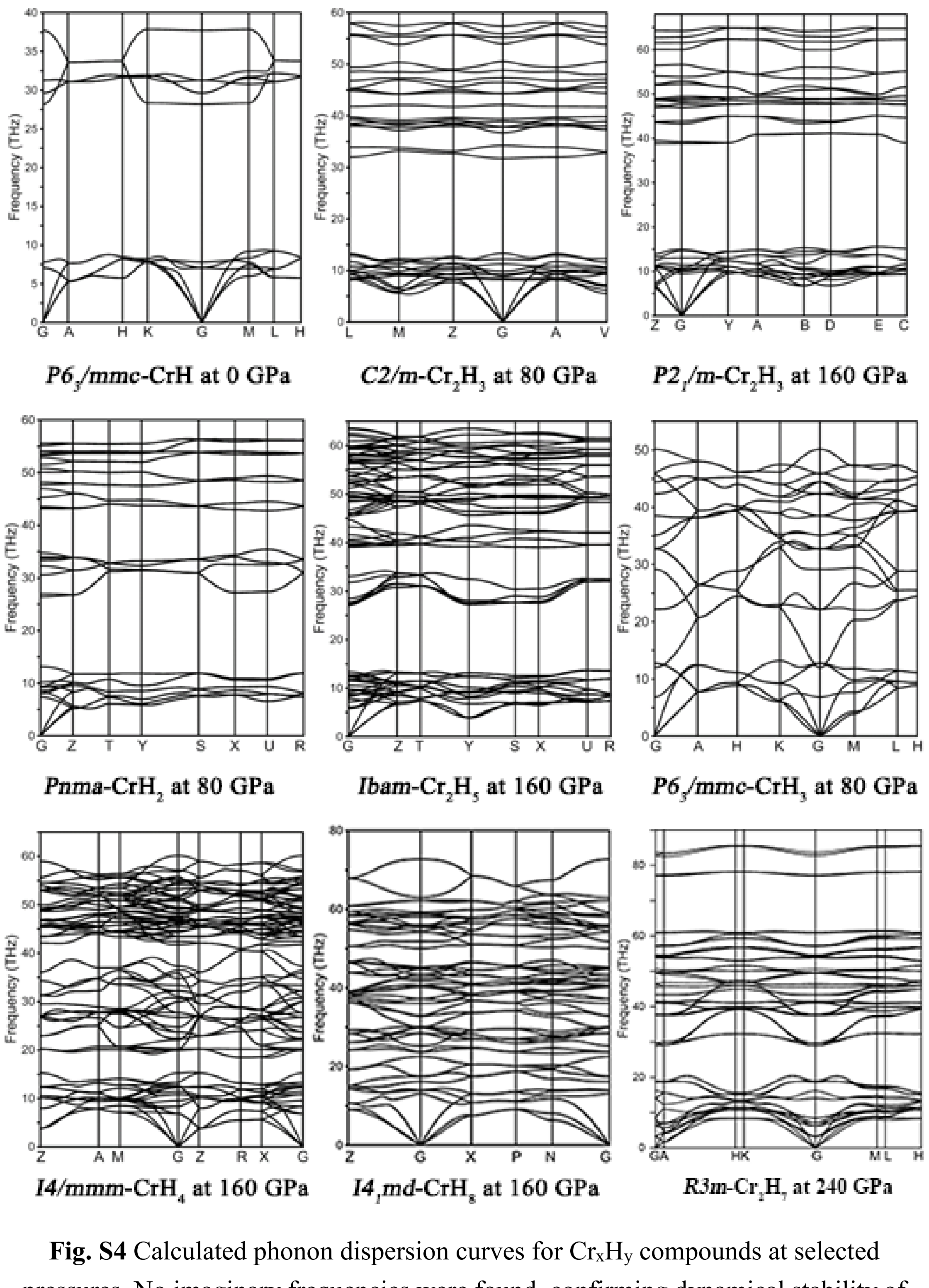

Fig. S4 Calculated phonon dispersion curves for Cr_xH_y compounds at selected pressures. No imaginary frequencies were found, confirming dynamical stability of these phases.

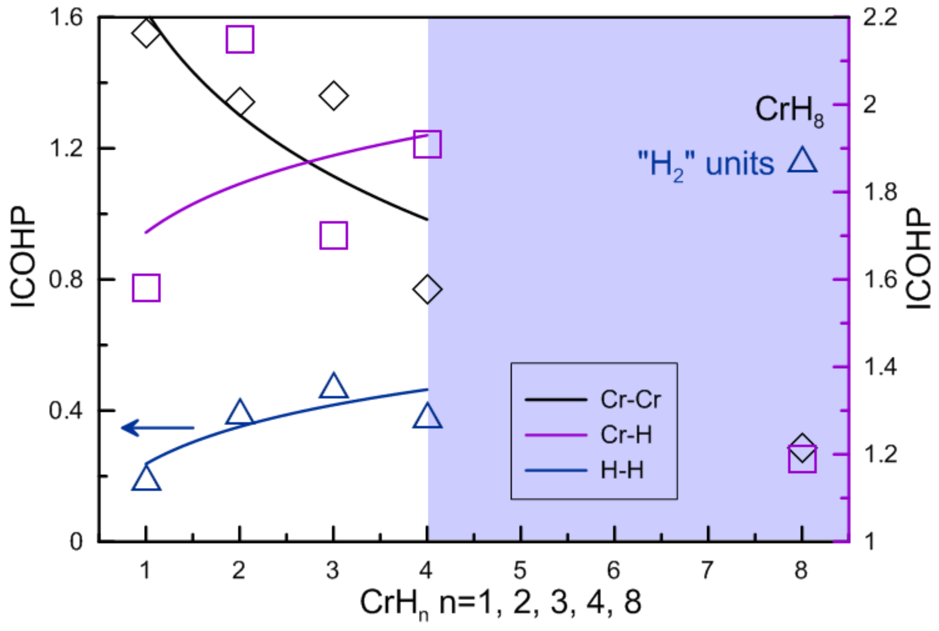

Fig. S5 (Color online) The calculated integrated crystal orbital Hamilton population (ICOHP) for Cr-Cr, Cr-H and H-H interactions in CrH_8 at 160 GPa.

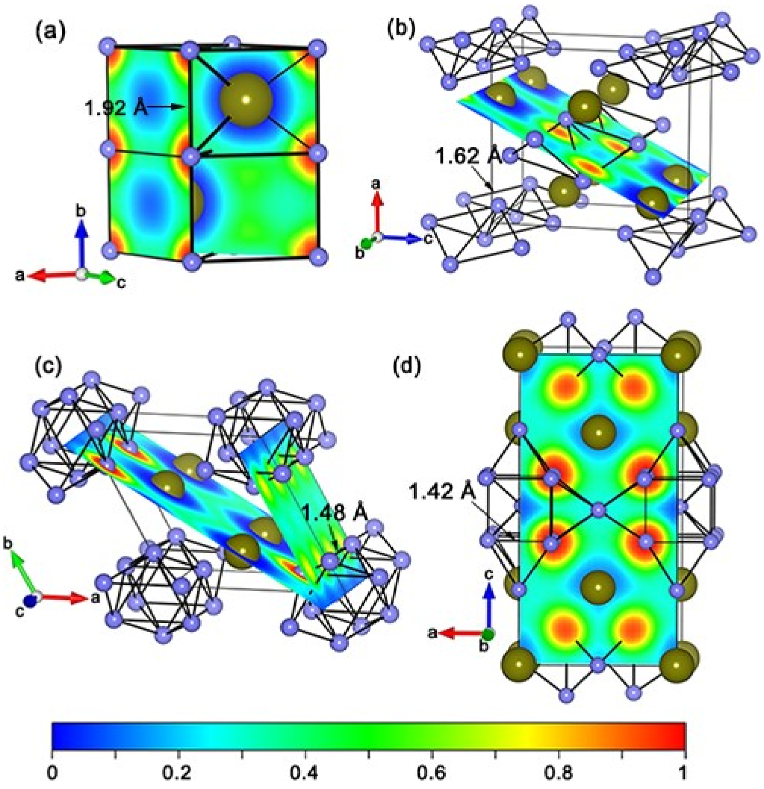

Fig. S6 (Color online) Electron localization function (ELF) distributions for stable CrH_n (n=1-4) compounds at 200 GPa. There are large ELF values between Cr and H atoms, suggesting relatively strong covalent component of bonding.

In order to check the relative stability of $CrH_{0.97}$, we have built a 3*3*2 supercell of CrH with a single H vacancy, i.e. $Cr_{36}H_{35}$. Then, we consider the temperature dependence of free energy of the suggested chemical reaction $Cr_{36}H_{35}$ (s) + 0.5 H_2 (g) = 36 CrH (s) by applying the following method.

A. Free energy of solids (CrH and $CrH_{0.97}$)

The free energy of solid (CrH and $CrH_{0.97}$), can be expressed as,

$$G(T,V) = U(V) + F_{elec} + F_{vib} + F_c(T) \qquad [1]$$

where U is the internal lattice energy, while F_{elec}, F_{vib} and F_c denote the electronic, vibrational and configurational energies, respectively. The electronic excitations are usually neglected, while vibrational contributions represents the phonon energy, which can be treated in the harmonic approximation, by summing over the normal modes frequencies,

$$F_{vib} = \frac{1}{2}\sum_{i=1}^{n} hv_i [\frac{1}{2} + \frac{1}{\exp(-hv_i / kT) - 1}] \qquad [2]$$

where k is Boltzmann constant, h is Planck constant.

B. Free energy of H_2 gas

On the other hand, the free energy of diatomic H_2 gas molecule can be expressed as:

$$G(H_2) = U(H_2) + \frac{7}{2} k_B T - TS_2 \qquad [3]$$

where the entropy S can be divided into four parts, namely, translation entropy S_{trans}, rotational entropy S_{rot}, vibrational entropy S_{vib} and electronic entropy S_{elec}. Again, S_{elec} is neglected, while the other terms can be defined as,

$$S_{trans} = \frac{5}{2}Nk + Nk\ln[\frac{V}{N}(\frac{2\pi mkT}{h^2})^{3/2}] \qquad [4]$$

$$S_{rot} = Nk + Nk\ln\frac{T}{2\theta_{rot}} \qquad [5]$$

$$S_{vib} = \frac{Nk\theta_{vib}(\frac{1}{2} + \frac{1}{\exp(\theta_{vib}/T) - 1})}{T} + Nk\ln(\frac{\exp(-\theta_{vib}/T)}{1 - \exp(-\theta_{vib}/T)}) \qquad [6]$$

Here, θ_{rot} and θ_{vib} are the characteristic rotational and vibrational temperatures, respectively.

$$\theta_{rot} = \frac{(h/2\pi)^2}{2kI} \qquad [7]$$

$$\theta_{vib} = \frac{hv}{k} \qquad [8]$$

where I is a molecular moment of inertia, v is the vibration frequency of H_2 molecule. To be consistent with the free energy calculation of solids, here we calculated the entropy of H_2 within the same functional. The relaxed H-H bond length in H_2 molecule is 0.74 Å, in satisfactory agreement with the experimental report (0.741 Å).

The theoretical vibrational frequency for H-H stretching modes of H_2 is 46.8 THz, compared with the experimental value 45.77 THz. Note that the vibrational component of the gas entropy here is very small (less than 5% of the total). The calculated temperature dependent $S(H_2)$ is shown in Fig. S7. Compared to the experimental value ($S_{expt}(H_2)$ = 130.68 J/(mol · K)) at standard conditions, our results are close.

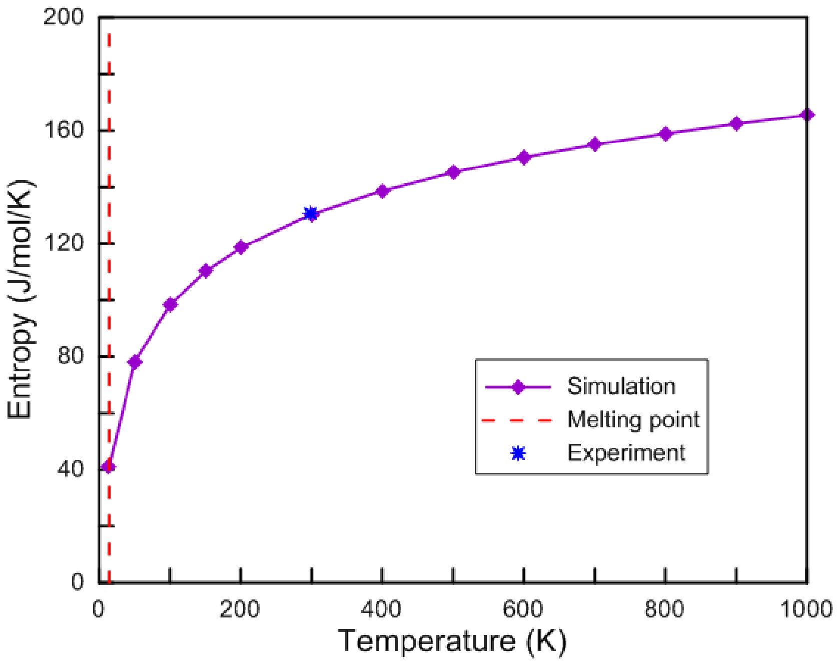

Fig. S7 (Color online) The calculated entropy of H_2 gas at different temperatures. The melting point (14.01 K) is also shown for being consistent.

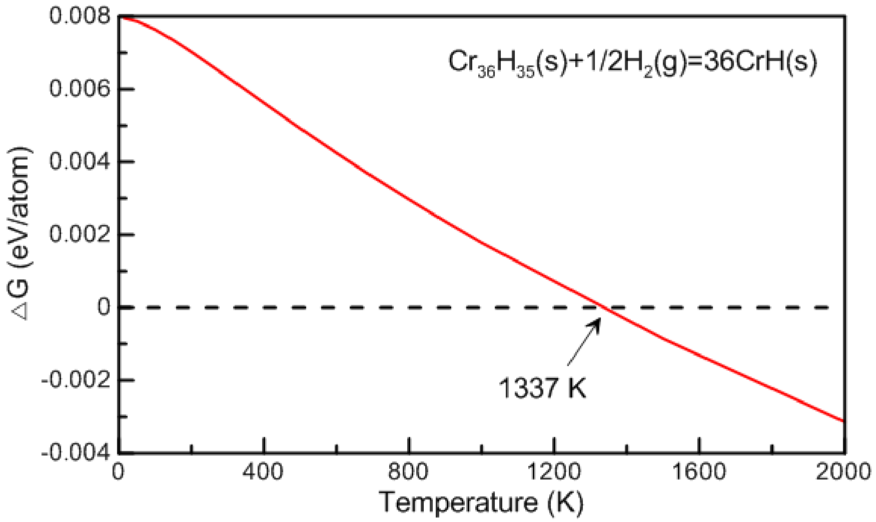

Fig. S8 (Color online) The temperature dependence of free energy of the suggested chemical reaction $Cr_{36}H_{35}$(s) + 0.5H_2(g) = 36CrH(s).

By applying the eq. 1 and eq. 3, we obtain the formation energy of the suggested chemical reaction $Cr_{36}H_{35}$ (s) + 0.5 H_2 (g) = 36 CrH (s). The results are shown in Fig. S8. The predicted decomposition temperature is 1337 K. The temperature is easily

accessible, thus these materials are promising to synthesize. At low temperature, the free energy of this reaction is negative, i.e. CrH is more stable than $Cr_{36}H_{35}$+0.5 H_2. As you increase temperature, right-hand side of the reaction will become less and less favorable. At 1337 K, $Cr_{36}H_{35}$ (i.e. $CrH_{0.97}$) becomes more stable.

Tab. S4 Calculated λ, ω_{log} and T_c values for CrH and CrH_3 using $\mu^* = 0.1$ and 0.13 at selected pressures.

Phase	Pressure (GPa)	λ	ω_{log}	T_c (K) μ*=0.1	T_c (K) μ*=0.13
CrH	0	0.67	338.5	10.6	7.9
	60	0.48	415.5	4.3	2.5
	120	0.43	531	3.3	1.6
	200	0.42	560	3.1	1.5
CrH_3	81	0.95	568.1	37.1	30.9
	120	0.8	630.5	29.5	23.9
	160	0.76	670.6	28.2	22.4
	200	0.69	810	27.2	20.7

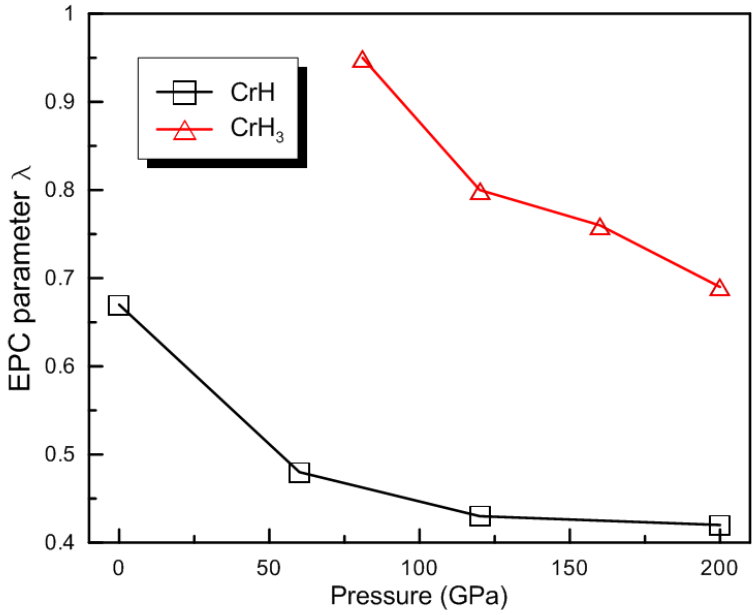

Fig. S9 (Color online) The electron-phonon coupling (EPC) parameter λ as a function of pressure for CrH and CrH_3.

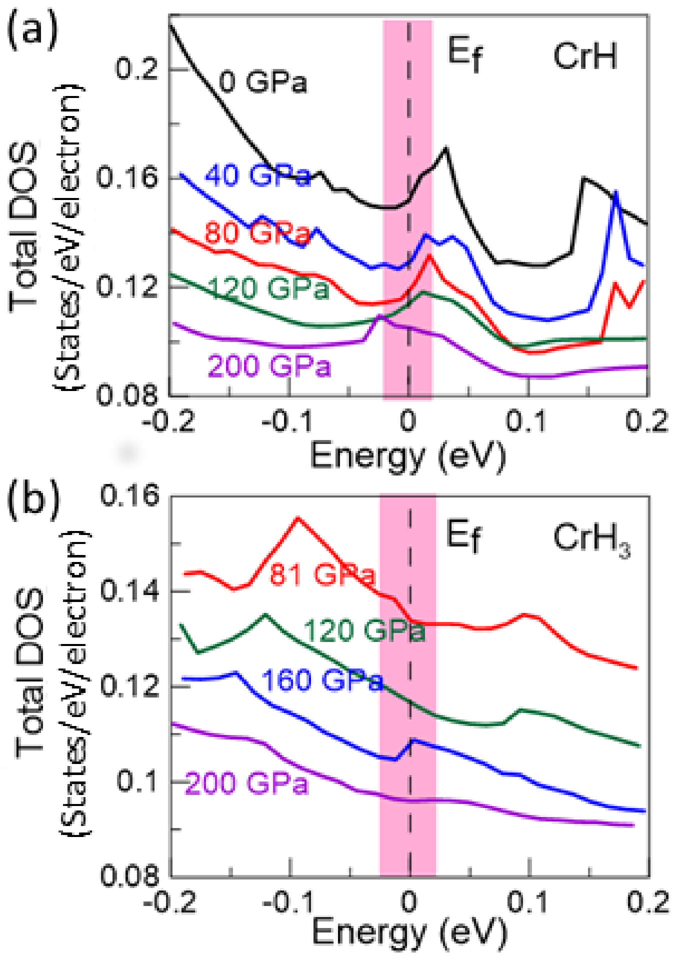

Fig. S10 (color online) The total electronic densities of states around Fermi level for (a) CrH and (b) CrH_3 at selected pressures.

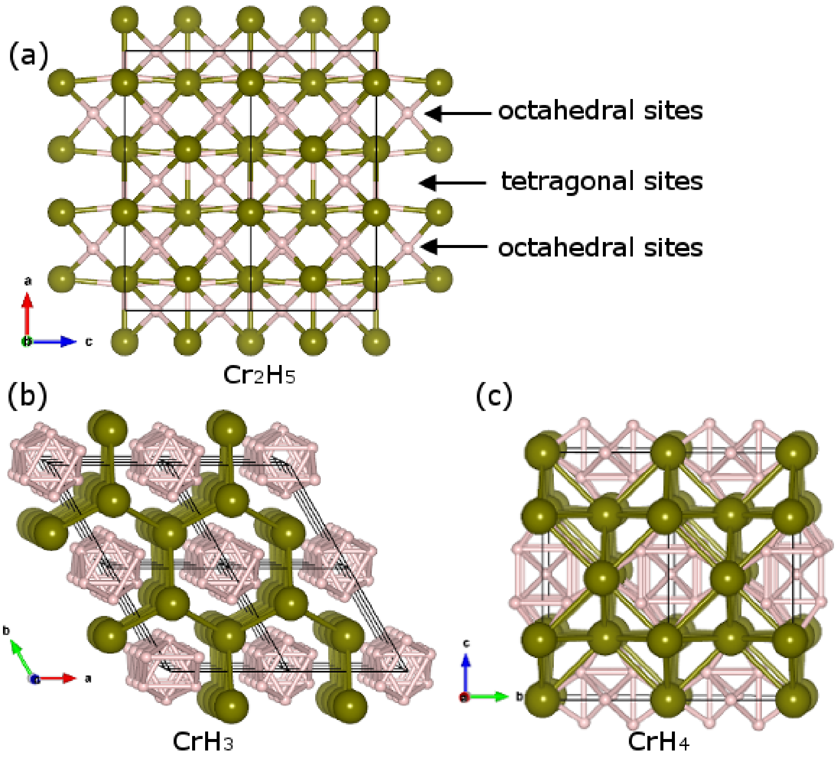

Fig. S11 (color online) Predicted ground-state static structures of (a) Cr_2H_5 (*Ibam* at 160 GPa), (b) CrH_3 (*P6₃/mmc* at 160 GPa) and (c) CrH_4 (*I4/mmm* at 160 GPa). Octahedral and tetrahedral sites of distorted *hcp* Cr sublattice are indicated in panel a. In panels b and c, yellow lines indicate Cr-Cr separations shorter than 2.6 Å, white lines indicate H-H separations shorter than 1.6 Å. The highlighted host-guest structures of CrH_3 and CrH_4.
